# Body Awareness: Construct and Self-Report Measures

**DOI:** 10.1371/journal.pone.0005614

**Published:** 2009-05-19

**Authors:** Wolf E. Mehling, Viranjini Gopisetty, Jennifer Daubenmier, Cynthia J. Price, Frederick M. Hecht, Anita Stewart

**Affiliations:** 1 Osher Center for Integrative Medicine, University of California San Francisco, San Francisco, California, United States of America; 2 Department of Family and Community Medicine, University of California San Francisco, San Francisco, California, United States of America; 3 Department of Medicine, University of California San Francisco, San Francisco, California, United States of America; 4 Department of Psychosocial and Community Health Nursing, University of Washington, Seattle, Washington, United States of America; 5 Department of Social and Behavioral Sciences, Institute for Health & Aging, University of California San Francisco, San Francisco, California, United States of America; University of Granada, Spain

## Abstract

**Objectives:**

Heightened body awareness can be adaptive and maladaptive. Improving body awareness has been suggested as an approach for treating patients with conditions such as chronic pain, obesity and post-traumatic stress disorder. We assessed the psychometric quality of selected self-report measures and examined their items for underlying definitions of the construct.

**Data sources:**

PubMed, PsychINFO, HaPI, Embase, Digital Dissertations Database.

**Review methods:**

Abstracts were screened; potentially relevant instruments were obtained and systematically reviewed. Instruments were excluded if they exclusively measured anxiety, covered emotions without related physical sensations, used observer ratings only, or were unobtainable. We restricted our study to the proprioceptive and interoceptive channels of body awareness. The psychometric properties of each scale were rated using a structured evaluation according to the method of McDowell. Following a working definition of the multi-dimensional construct, an inter-disciplinary team systematically examined the items of existing body awareness instruments, identified the dimensions queried and used an iterative qualitative process to refine the dimensions of the construct.

**Results:**

From 1,825 abstracts, 39 instruments were screened. 12 were included for psychometric evaluation. Only two were rated as high standard for reliability, four for validity. Four domains of body awareness with 11 sub-domains emerged. Neither a single nor a compilation of several instruments covered all dimensions. Key domains that might potentially differentiate adaptive and maladaptive aspects of body awareness were missing in the reviewed instruments.

**Conclusion:**

Existing self-report instruments do not address important domains of the construct of body awareness, are unable to discern between adaptive and maladaptive aspects of body awareness, or exhibit other psychometric limitations. Restricting the construct to its proprio- and interoceptive channels, we explore the current understanding of the multi-dimensional construct and suggest next steps for further research.

## Introduction

In recent years, a construct labeled “body awareness” has emerged as a subject of scientific research across a wide range of health topics. Although a clear definition is rarely provided [1], [2], body awareness involves an attentional focus on and awareness of internal body sensations. The term has traditionally been used in studies of anxiety and panic disorders to describe a cognitive attitude characterized by an exaggerated patient focus on physical symptoms, magnification (“somatosensory amplification”), rumination, and beliefs of catastrophic out-comes [3]. In this conceptualization of body awareness, the number of perceived and presumed potentially distressing body sensations has been widely used as a marker for hypochondriasis, anxiety and somatization [3], all strongly associated with unfavorable clinical outcomes such as the trajectory of pain [4]. Accordingly, the dominant view in medical and behavioral science considers heightened awareness of somatic information as potentially distressing and maladaptive. There remains considerable concern among clinicians that efforts to enhance body awareness or to focus attention on body symptoms may lead to an obsession or undue dwelling on bodily functions, subsequently creating somaticizing “cripples” with anxiety and depression [5]–[7]. Consequently, when this understanding of body awareness is applied to studies of pain, for example, one would expect benefits from distraction, an attentional focus directed away from pain sensations and towards mental tasks, such as solving mathematical problems. Indeed, studies of experimental pain or other acute pain models demonstrate such benefits [8]–[10].

However, distraction from chronic pain during a pain-inducing activity is associated with greater post-activity pain [11]. Furthermore, recent studies have shown that somatosensory amplification, the tendency to experience normal bodily sensations as intense and noxious, does not reflect heightened sensitivity to bodily sensations [12]–[15]. Rather, subjects who experience body sensations of normal quality and intensity as intense and disturbing are less accurate in detecting subtle bodily sensations [12]–[14]. Thus, the ability to notice subtle bodily sensations can be viewed as a process distinct from somatosensory amplification. When body awareness is defined as the ability to recognize subtle body cues [5], preliminary evidence suggests that it may be useful in the management of chronic diseases such as chronic low back pain [16], [17], congestive heart failure [5], chronic renal failure [18], and irritable bowel syndrome [19]. Regarding pain research, studies using specific cognitive interventions provided by clinical psychologists, such as guided attention allocation to the sensory aspect of pain or sensory discrimination training, have shown significant benefits for patients with chronic pain [20], [21]. For example, in patients with chronic low back pain, a recent study confirmed that a focus on the sensory components of pain was more beneficial than attempts to suppress awareness of that pain [20]. Similarly, studies with patients suffering from phantom pain have shown that sensory discrimination training can reduce pain [21] and reorganize phantom pain-related representation areas of the sensory cortex.[22]. These findings seem to contradict the traditional understanding of body awareness and suggest that body awareness is a complex, multi-dimensional construct in need of more nuanced conceptualization.

Another construct related to body awareness is “body image”. “Image” implies that this aspect of body awareness includes an exteroceptive, visual channel of perception. This aspect is explored in a vast literature from psychiatry (i.e. anorexia) to feminist psychology (i.e. self-objectification) and neuroscience (i.e. rubber hand illusion, amputees) reflecting a preferential reliance on visual appearance over perceptions from inside the body [23]–[25]. An integration of all ramifications and aspects of body awareness from such disparate and rarely converging fields of discourse and research would constitute an intellectual challenge beyond the scope of our study. For the purpose of this paper we are primarily concerned with those aspects of inner body awareness that, although interacting with thoughts and exteroceptive stimuli, are distinguishable from these and are potentially of key relevance for a deeper understanding of the interaction of mind and body. Therefore, we limit our review and restrict our definition of body awareness to the core-awareness of sensations from inside the body and exclude exteroceptive channels.

From a neuro-physiological viewpoint, this definition of the core-construct of inner body awareness, though more narrowly defined, relates primarily to proprioception and interoception. Proprioception is the perception of joint angles and muscle tensions, of movement, posture and balance and has become an integral part of neuromuscular rehabilitation after injuries and of the prevention of falls in the elderly [26]. Objective measurement of proprioception requires sophisticated technical equipment with limited feasibility for a broader application in clinical settings outside the laboratory [26], [27]. Interoception is the perception of sensations from inside the body and includes the perception of physical sensations related to internal organ function, such as heart beat, respiration, satiety, and autonomic nervous system activity related to emotions [28]–[31]. Neuroscience has suggested a network of brain regions where interoception is processed, how it is related to emotions and pain, and how essential it is for decision making [32]–[35]. Awareness of internal physical sensations has been linked with activations in specific brain areas including the right anterior insula and cingulate cortices [36] and the pathways for a multi-level integrated representation of inner-body experience have been clarified [29], [37]. Awareness of physical sensations associated with emotions is a key element for affect regulation and for the sense of self [34], [38]–[40]. Inter-individual variations in interoceptive capacity have been found to be associated with right anterior insula activity [21], [32], and a meditative practice involving sustained mindful attention to internal (and external) sensory stimuli with right anterior insula cortical thickness [30], [41], and grey matter density [42] suggesting a potential, although still speculative, neuroplasticity effect due to meditation (practicing sustained attention to respiratory and other sensations) and interoceptive body awareness [32], [41].

To clarify our use of terms: Interoception is the processing of sensory input from inside the body in contrast to exteroception, the processing of input from outside the body (vision, hearing, smell, taste and touch, with touch and taste having components of both). Proprioception and interoception are terms of sensory perception, a complex process that includes the objective processes of neural coding, transduction and central representation of peripheral stimuli and, most importantly, entails both afferent (bottom-up) and efferent (top-down or gating) mechanisms. Much of this information is processed “before we know it”, pre-cognitive, unconsciously. Interoceptive information, for example, is not identical to interoceptive awareness. Some of this information can enter consciousness, and we become aware of it. Subjective awareness, in turn, is strongly influenced by mental processes including attention, interpretation, appraisal, beliefs, memories, conditioning, attitudes and affect. Much of perception research has been directed toward the study of either exteroception or pain. In both fields the involved mechanisms have long been acknowledged for their enormous complexity, and experimental research is beginning to uncover the complexity of interoception as well. Body awareness, as we define it here, is the subjective, phenomenological aspect of proprioception and interoception that enters conscious awareness, and is modifiable by noted mental activities.

How can we understand the construct of body awareness when paying increased attention to one's sensory features, be they appraised as comfortable or uncomfortable (i.e. pain), can be both adaptive and maladaptive? As chronic pain and depression are closely associated, findings from pain research are intriguingly similar to findings from depression research: self-awareness or the awareness of symptoms in depressed patients can be adaptive or maladaptive according to “distinct and incompatible modes of mind”; a ruminative self-focus appears to be maladaptive whereas focusing attention directly on immediately experienced feelings appears to be adaptive [43]. These distinct modes of attention or self-reference can be dissociated through attentional training and identified by their distinct neural activation and connectivity in the brain [44]. In pain research a parallel distinction between different modes of attention has been reported to be of prognostic key importance: Although pain seems to have an attention-redirecting function (from an external attention focus towards the pain region), hypervigilance is associated with worse chronic pain and seems to have a negative impact on cognitive function [45]. A diffuse, emotion-based hypervigilance seems to be maladaptive, whereas “concrete somatic monitoring” or “sensory discrimination” of the precise details and present-moment characteristics in physical sensations appear to be adaptive [3], [46]. However, the traditional view of the construct of body awareness does not account for these “distinct and incompatible modes of mind” [43] (or modes of attention). In the past, most research and clinical therapies were based on a conceptual understanding of body awareness that focused on the negative aspects of heightened body awareness as it overlaps with hypochondriasis and somatization. As new research suggests a potential value of interoceptive awareness of subtle bodily sensations, the traditional view of body awareness is challenged to recognize the complexity and ambiguity of this construct for psychosomatic research and therapies [47], [48].

Yet another perspective on body awareness comes from academic disciplines outside of medical and behavioral sciences: contemporary philosophers [49]–[51], anthropologists [52], and linguists [53] dedicate a growing body of literature to the theme of ‘embodiment’. Embodiment is understood as the felt sense of being localized within one's physical body [54] and references the lived immediate experience of one's own body [55], [56]. Overcoming the constraints of Cartesian dualism, embodiment recognizes the role that our body plays in shaping our thinking and culture [39], [50], [56]. “It has often been observed that modern Western society is typified by a certain ‘disembodied’ style of life…. A rising interest in finding ways to ‘return to the body,’ whether via exercise, Hatha yoga, body therapies, craft-work, or intimacy with nature, is but a reaction to this general trend toward a ‘decorporealized’ existence” [57]. The growing public interest in methods for stress reduction that use interoceptive awareness i.e. of sensations related to respiration [58]–[60] has led to a fascinating discourse among neuro-scientists, philosophers and spiritual teachers [61], [62] regarding the relationship of mind and body. Neurobiologist Edelman states: “Consciousness is embodied” [56]. Going beyond Descartes' “cogito ergo sum” [63], a distinction is emphasized between thinking about the body and an ‘embodied presence’ in the body [64], a quality of immediate present-moment perception barely altered by beliefs and appraisal [65]. From contemplative traditions, stress reduction methods borrow the practice of a particular attentional focus on subtle physical sensations, such as breathing, in order to relax into an ‘embodied’ awareness of mind-body integration [66] with early evidence of some health benefits [67]. It is precisely this mental movement beyond rational or irrational thinking about physical symptoms (interpreting, appraising and eventually ruminating with fearful hypervigilance) to a meta-cognitive (controlled and monitored) state of sustained present-moment attention to events within the body, often labeled as mindfulness [43], [65], [68], that is both the subject of philosophical discourse and a particular quality of inner body awareness [64].

Attempts to define, operationalize and measure the construct of ‘mindfulness’ have been facing challenges similar to body awareness [69]. How does body awareness relate to mindfulness? The most comprehensive measure of mindfulness is based on a five-factor model with the following labels: 1) Non-reactivity to inner experience; 2) observing/noticing/attending to sensations/perceptions/thoughts/feelings; 3) Acting with awareness/automatic pilot/concentration/nondistraction; 4)describing/labeling with words; and 5) nonjudging of experience [70]. Close observation of internal experience was defined as awareness of internally generated stimuli, such as sensations, cognition, and emotions. Thus, mindfulness encompasses more than awareness of inner sensations by including awareness of cognitive thoughts of any kind, which are not excluded from the body awareness construct [71]. Moreover, the mindfulness facet of ‘Observing’ does not explicitly separate attention to internal (thoughts, feelings, sensations) from attention to external stimuli, such as sights, sounds, and smells [70]. Thus, the scope of awareness is more narrowly defined in the construct of body awareness compared to mindfulness. However, mindfulness skills (sustained attention, concentration, non-reactivity, nonjudging of experience) are expected to play a major role in the shaping of body awareness.

A variety of therapeutic approaches in common use throughout the world claim to enhance body awareness [72] including yoga [23], [73], TaiChi, massage [74]–[76], Body-Oriented Psychotherapy [74], mindfulness based therapies/meditation [41], Feldenkrais [77], Alexander Method [78], Breath Therapy [79], and even mental training for athletic exercise and sport performance [80]–[82]. These approaches are often categorized as mind-body approaches and/or manual therapies [83] and enjoy a growing popularity in the Western world [59] but frequently suffer from a lack of theory and methodologically weak research behind esoteric formulations and unfounded statements of benefits. Related therapeutic approaches offered by physical therapists in Sweden, Norway and the Netherlands explicitly carry names such as Body Awareness Therapy (BAT) or Body Awareness Program (BAP) [84], [85]. Generally speaking, all of these approaches aim to cultivate a particular quality of body awareness characterized not by its intensity (exaggerated or ignored) but by non-judgmental ‘mindfulness’, “a quality of non-elaborative awareness to current experience and a quality of relating to one's experience with an orientation of curiosity, experiential openness, and acceptance” [65]. By today, they have been studied to a preliminary degree in patients with a variety of medical conditions including chronic low back pain [16], [86]–[89], pelvic pain [2], [90], fibromyalgia [91]–[93], musculoskeletal pain [94], [95], chronic pain in general [95], [96], disordered eating and obesity [23], [97], [98], irritable bowel syndrome [99], sexual abuse trauma [74], [100], coronary artery disease [101], [102], congestive heart failure [5], chronic renal failure [18], falls in the elderly [103], anxiety [104], [105] and depression [106]. In order to determine whether body awareness indeed plays a role in these clinical areas and therapeutic approaches, we need a more precise understanding and reliable, valid measurement of this construct.

Cameron stated: “Bodily awareness is essential to the concept of self” [107]. Although there is a great need to study practices of interoception and body awareness and their potential clinical benefits, few attempts have been made to measure changes in body awareness associated with such interventions [108], and to link intervention-related changes in body awareness to clinical outcomes.

Considerable research effort is underway to illuminate proprioceptive and interoceptive processes and their neural basis. This research is generally conducted in laboratories assessing singular perception modalities, such as perception of heart rate, gastric motility, respiratory load, joint angles, muscle tension and others. The topic of body awareness is further complicated by the fact that individuals do not experience atomistic “sensations”, e.g. complaining about a painful sensation in the neck, but, even within one modality, rather “perceptual wholes” in which bodily sensations are integrated into “gestalts” that also include affect, intention, values etc. Present moment, immediate experience is habitually integrated with narrative self-reference linking present with past experiences across time [109]. Interoceptive afferents within uni-modular sensory systems are centrally integrated into a larger neural system that has been termed the Homeostatic Interoceptive System [29], [110]. There is very preliminary experimental support for the notion that interoceptive accuracy might have trait and state aspects that co-vary across modalities [111], [112] reflecting a general sensitivity for visceral processes. Objective measures (discussed below) allow for experimental studies but are restricted to laboratory settings and reflect singular aspects of a person's complex experience.

We feel that this research field could benefit from a multi-modal self-report measure that could be used with experimental protocols as well as in clinical settings and that could potentially discriminate between beneficial and maladaptive types of body awareness. Numerous self-report instruments of body awareness have been developed that exclusively measure anxiety related symptoms [113]–[116]. Newer instruments reflect an increasingly complex conceptualization of body awareness [23], [117]–[119]. The purpose of this paper is to answer the following questions: Are we able to appropriately measure this ambiguous construct of body awareness by self-report in a clinical context outside of a laboratory? And, how is the construct of body awareness understood in existing measures?

We conducted a systematic review of self-report instruments attempting to measure body awareness with two aims: a) to review the instruments' psychometric properties and b) to further examine the understanding of the construct that underlies the various instruments and their dimensions queried. The review was used to explore the current understanding of the construct, to support this expanding field of research and to suggest next steps for further research development.

As a starting point, a multidisciplinary group of researchers (Katrina Carlsson; Jennifer Daubenmier; Eric Jacobson; Janet Kahn; Catherine Kerr; Wolf Mehling; Cynthia Price; Stephanie Shields; Jim Stephens.) engaged in research related to the construct developed a working definition of body awareness:

Body awareness is the perception of bodily states, processes and actions that is presumed to originate from sensory proprioceptive and interoceptive afferents and that an individual has the capacity to be aware of.

Body awareness includes the perception of specific physical sensations (e.g., awareness of heart activity; proprioception of limb position) as well as complex syndromes (e.g., pain; sense of relaxation; ‘somatic markers’ of emotions).

Body awareness is hypothesized as the product of an interactive and dynamic, emergent process that a) reflects complex afferent, efferent, forward and back-projecting neural activities, b) includes cognitive appraisal and unconscious gating, and c) is shaped by the person's attitudes, beliefs, experience and learning in a social and cultural context.

Our definition attempts to integrate some of the above summarized research and perspectives from primary care medicine, behavioral science, health psychology, cognitive neuroscience, anthropology, massage therapy, physical therapy, body-oriented psychotherapy, martial arts, Yoga, Feldenkrais, breath therapy and Rolfing.

## Methods

**Figure 1:**
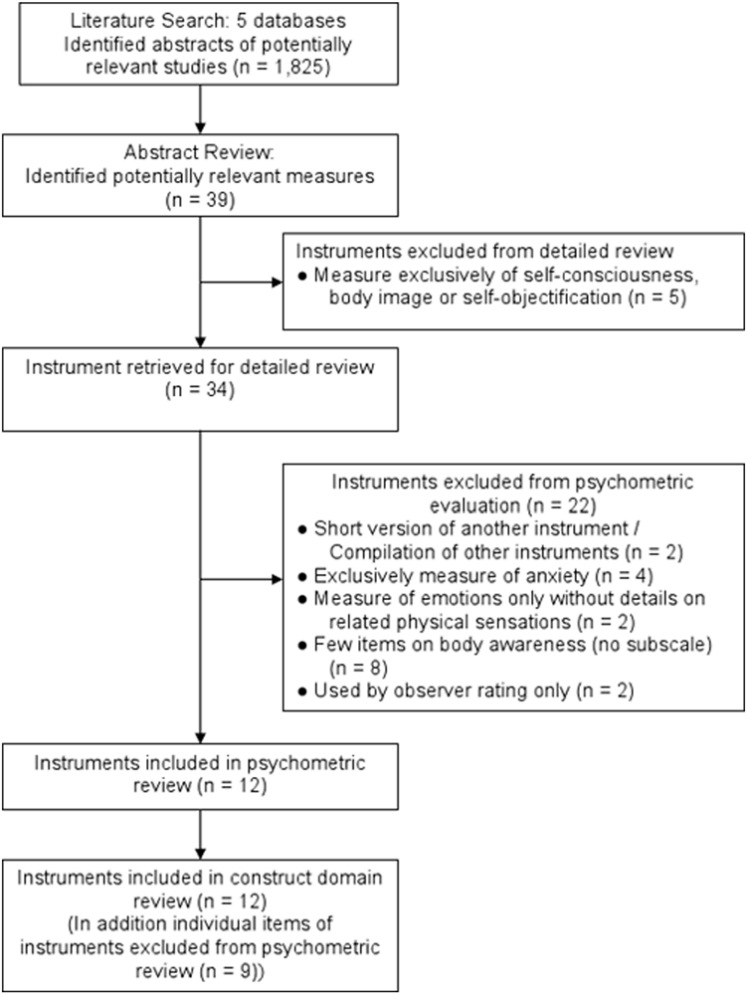
Review Steps.

Figure 1 outlines the sequence of steps for this review. In April and May 2007 we conducted a systematic search of 5 electronic databases to identify articles reporting the use of body awareness-related instruments: PubMed, PsycInfo, EMBASE, HAPI, and the Digital Dissertations Database. Search terms included the following MeSH headings, keywords, descriptors, and text words in titles and abstracts: “body awareness”, “body perception”, “body sensation”, “body consciousness”, “movement or perception and awareness”, “sensation and awareness”, “interoception or introception”, and “physical sensation”. These search terms were combined with singular and plural terms for instruments: “questionnaire”, “scale”, “rating”, “instrument”, and “inventory”. No limits were placed on language, country or time of publication. Publications containing the terms “body image” and “self image” were specifically excluded from the search as according to our working definition these terms are distinct from our restricted core-concept of body awareness.

### Systematic Review Step 1: Screening of Abstracts

In a first review step, the abstracts of all identified publications were screened for relevance by two reviewers (WM and VG). All abstracts that mentioned instruments described as measuring body awareness or perception/awareness of physical sensations were identified, and the full articles and instruments were obtained. We included instruments measuring physical sensations related to emotions but excluded instruments that referred to mood and emotions exclusively with verbal labels (such as anger, sadness, joy…) without any reference to physical sensations (such as feeling hot, tense muscles, a deep breath etc) (Table 1). We excluded instruments exclusively concerned with “body image” in teenagers or self-objectification in women. If an instrument that was claimed to measure a construct different from body awareness (i.e. mindfulness) contained items of body awareness-related aspects but did not have a separable subscale, it was excluded from the psychometric review (Table 1). However, these items were included in the conceptual review (Table 2).

**Table 1:** Instruments Excluded from Psychometric Review and Reason for Exclusion.

Author(s)	Name of Instrument	Acronym	Reason for Exclusion
Austin DW, Richards JC, Klein B [158]	Modified Body Sensations Interpretation Questionnaire	MBSIQ	7
Baer RA, Smith GT, Allen KB [117]	Kentucky Inventory of Mindfulness Skills	KIMS	5
Barsky AJ, Wyshak G, Klerman GL [122]	SomatoSensory Amplification Scale	SSAS	5
Bernet M [159]	Styles in the Perception of Affect Scale	SIPOAS	4
Brahler E, Strauss B, Hessel A, Schumacher J [160]	Fragebogen zur Beurteilung des eigenen Körpers	FBeK	7
Brown KW, Ryan RM [126]	Mindful Attention Awareness Scale	MAAS	5
Chambless DL, Caputo GC, Bright P, Gallagher R [113]	Body Sensations Questionnaire	BSQ	2
Clark DM, Salkovskis PM, Breitholtz E, et al [161]	Body Sensation Interpretation Questionnaire	BSIQ	5
Clark DM, Salkovskis PM, Breitholtz E, et al [161]	Brief Body Sensation Interpretation Questionnaire	BBSIQ	1
Deusinger I [162]	Frankfurter Koerperkonzept Skalen	FKKS/FBCS	3
Fenigstein A, Scheier MF, Buss AH [163]	Private and Public Self-Consciousness Questionnaire	PPSC	3
Fisher S [164]	Fisher Body Focus Questionnaire	BFQ	7
Fredrickson BL, Roberts T-A, Noll SM, Quinn DM, Twenge JM [165]	Self-Objectification Questionnaire	SOQ	3
Friis S, Skatteboe UB, Hope MK, Vaglum P [127]	Body Awareness Rating Scale	BARS	6
Garner DM [166]	Eating Disorder Inventory	EDI-C	5
Gisbers van Wijk CMT, Kolk AM [167]	Somatic Awareness Questionnaire	SAQ	1
Hart EA, Leary MR, Rejeski, WJ [168]	Social Physique Anxiety Scale	SPAS	3
Hoyer J [169]	Fragebogen zur dysfunktionalen und funktionalen Selbstaufmerksamkeit	DFS	5
Kawano R [170]	The Self Awareness Questionnaire	SAQ	5
Main CJ [114]	Modified Somatic Perception Questionnaire	MSPQ	2
Mandler G, Mandler JM, Uviller ET [115]	Autonomic Perception Questionnaire	APQ	2
McKinley NM, Hyde JS [171]	Objectified Body Consciousness Scale	OBCS	3
Pauluus P [172]	Fragebogen zum Korpererleben	FKE	7
Pekala RJ, Levine RL [173]	Phenomenology of Consciousness Questionnaire	PCQ	4
Roxendal G [128]	Body Awareness Scale-Health	BAS-H	6
Schneider A, Lowe B, Streitberger K [174]	Perception of Bodily Sensations Questionnaire	PBSQ	5
Stern RM, Higgins JD [116]	Somatic Perception Questionnaire	SPQ	2

Reason for Exclusion (Frequency).

1: Short version of another instrument/Compilation of other instruments (2).

2: Exclusively measures anxiety (4).

3: Measure of self-consciousness, body image or self-objectification (5).

4: Measures emotions only without details on physical sensations (2).

5: No or only few items on body awareness (8).

6: Uses observer rating only (2).

7: Instrument not obtainable (4).

**Table 2:** Instruments with additional individual body awareness-related items included in conceptual review.

Author(s)	Name of Instrument	Acronym	Number of Items
Baer et al. [117]	Kentucky Inventory of Mindfulness Skills	KIMS	7
Baer et al. [125]	Five-Facets Mindfulness Questionnaire	FFMQ	5
Barsky et al. [122]	Somato-Sensory Amplification Scale	SSAS	3
Bernet M [159]	Styles in the Perception of Affect Scale	SIPOAS	10
Brown, Ryan [126]	Mindful Attention Awareness Scale	MAAS	1
Clark et al. [161]	Body Sensation Interpretation Questionnaire	BSIQ	8
Garner DM [166]	Eating Disorder Inventory	EDI-C	3
Kawano R [170]	The Self Awareness Questionnaire	SAQ	5
Mandler et al. [115]	Autonomic Perception Questionnaire	APQ	10
Schneider et al. [174]	Perception of Bodily Sensations Questionnaire	PBSQ	5

### Systematic Review Step 2: In-depth Instrument Review

For the second review step, all instruments identified in the first step were anonymized, and the original items were independently reviewed by two reviewers (WM and VG). We used the criteria from step1 and the following exclusion criteria: instruments exclusively developed as proxy measures of anxiety, instruments measuring emotions exclusively without related physical sensations, instruments exclusively using observer ratings, and instruments not obtainable despite repeated attempts to contact the authors. Several instruments for anxiety include self-reported body sensations and symptoms, are in wide use and well validated, such as the Beck Anxiety Inventory (BAI [120]), the Hamilton Anxiety Rating Scale (HAM-A [121]), and the Somato-Sensory Amplification Scale (SSAS [122]). These three measures, 13 of 21, 8 of 14 and 6 of 10 items, respectively, refer to somatic symptoms seen as commonly associated with anxiety. A comprehensive review of all emotion-related measures would go beyond the scope of this article. When initial consensus about an instrument could not be reached, the opinion of a third reviewer (JD) was sought and discussed until unanimous agreement was reached. For instruments or subscales of instruments fulfilling our full inclusion criteria, we retrieved all related publications that provided data about their development, reliability or validity and contacted the authors for additional unpublished data.

### Psychometric Assessment

For the first aim of our study, the psychometric assessment, two reviewers (VG, WM) independently compared the psychometric characteristics of the reviewed measurements until consensus was reached in all categories. Table 3 explains the rating adapted from McDowell [123] and applied in Table 4. We followed the definitions of McDowell [123]. We understand reliability as “the consistency or stability of the measurement process across time and patients” most frequently assessed by internal consistency (expressed as Cronbach's coefficient alpha which reflects the intercorrelation between items *and* the number of items) and by test-retest repeatability (expressed as Pearson or rank-order coefficients or as intra-class correlation). Content validity refers to comprehensiveness or to how adequately the items cover the themes specified in the conceptual definition of its scope; this can be assessed by focus groups and in-depth cognitive interviews. The latter methods may be considered to be part of the systematic development and are reported in that context. A “gold standard” for assessing criterion validity in measures of body awareness does not exist. For such an abstract construct, validation of a measure involves a series of steps beginning with a conceptual definition of the construct that is revised as additional evidence accrues. A construct definition should indicate the internal structure of its components and the way it relates to other similar or discriminant constructs. Construct validation can only be established incrementally and is a continuing process aiding in our understanding and revision of the construct [124]. Coefficients of correlations with data from other constructs are interpreted against a-priori hypotheses; exploratory and confirmatory factor analyses clarify the clustering of items and their representation of a process model of the construct; and assessments of the measure's ability to detect change and expected between-group differences validate the performance of a measure. For our rating of the reviewed instruments we followed the instructions suggested by McDowell for summary tables [123]. For the quality rating of the reviewed instruments we added two criteria: Increasing body awareness has been suggested as a potential mechanism of action for the benefits from certain therapies for patients with chronic pain; therefore we wanted to see whether any instruments were used to measure pain-related body awareness. In addition, we felt that measures that provide a detailed description of their systematic development should be acknowledged.

**Table 3:** Criteria for Reliability and Validity Rating (modified after McDowell, 2006).

**How widely the instrument has been used**	“few”: 1–4 published studies;
(refers to the number of separate studies in which the instrument was used. Studies were identified through the Web of Science Database and Google Scholar and by correspondence with the authors)	“several”: 5–12 studies by different groups;
	“many”: >12 studies.
**Thoroughness of reliability testing:**	“0”: no reported evidence of reliability;
	*: basic information only by the original author;
	**: several types of tests reported by the original author;
	***: several types of tests reported by different authors from separate studies;
	****: all major forms of reliability were reported in numerous studies.
**Results of the reliability testing**	“0”: no numerical results reported;
	*: evidence suggesting lack of substantial reliability (alpha<.80);
	**: adequate reliability (alpha≥.80);
	***: adequate reliability confirmed in independent sample by different research group;
Note: Instruments received an additional * for test-retest >.60 when otherwise rated as * or **.	****: excellent reliability: higher coefficients than those normally seen in other instruments.
**Thoroughness of validity testing:**	“0”: no reported evidence of validity;
	*: basic information only by the original author;
	**: several types of tests but reported results only by original author;
	***: several types of tests *and* reported results by different authors from separate studies;
Note: The reporting of Pearson correlation coefficients without a-priori qualification of the expected range of correlation justified only a rating as “basic information”.	****: all major forms of validity were reported in numerous studies.
**Results of validity testing:**	“0”: no numerical results reported;
	*: evidence suggesting weak validity;
	**: adequate validity;
	***: adequate validity confirmed by separate research group in separate sample;
Note: The instrument received an additional * for proven sensitivity to change and/or ability to distinguish between known groups when otherwise rated as * or **.	****: excellent validity: higher coefficients than those normally seen in other instruments.
**Population the instruments was used in:**	“0”: no patients;
	*: non-pain patients;
	**: both non-pain and pain patients.
**Evidence of systematic construct and/or instrument development:**	“0”: not reported;
Note: additional stars to a maximum of *** for: systematic focus group (report of systematic qualitative process to evaluate focus group statements), cognitive testing (report of systematically assessing understanding and interpretation of items), or item selection by factor analysis from a larger item pool.	*: based on a review of limitations of prior instruments

**Table 4:** Psychometric Evaluation of Body Awareness Instruments.

Name of measure or instrument	BIS	BRQ	BAM	TQ	SBA	QCC	PBCS	BPQ Sub-scales	SBC	BVS	BAQ
Scale	ordinal	interval	interval	interval for 3 nominal for 1	ordinal	continuous (VAS)	interval	ordinal	ordinal	interval	interval
Number of Items	31	7	13	4	4	30	5	82	20	4	18
Application	research	research	research	research	research	research	research	research	clinical/research	clinical/research	research
Studies using Measure	few	few	few	Few	few	few	many	few	few	few	many
Reliability: Thoroughness	*	*	*	0	**	*	**	0	*	***	***
Reliability: Results	** (α .89 for total scale)	** (two of 3 reported α values>.80)	**	0	** (No 3^rd^ * for test-retest, no 2^nd^ author)	* (2 of 3 subscales had alpha<.80)	** (α<.80 but Test-Retest r>.60)	0	** for BA * for BD subscales	*** (Some values borderline)	****
Validity: Thoroughness	0	**	*	*	*	*	****	0	**	***	***
Validity: Results	0	** (known gps+; Sens to change)	*	*	**	*	***	*	*** (Sens to chg+; FA+; Eating+)	***	*** (Most data from author, no 2^nd^ FA)
Used in patients/patients with pain	0	0	0	0	*	0	**	0	*	*	*
Concept development	***	*	**	*	*	*	*	0	***	*	***

BIS: Body Intelligence Scale by Anderson, 2006 [118].

BRQ: Body Responsiveness Questionnaire by Daubenmier J, 2005 [23].

BAM: Body Awareness Measure by Forester CA, 2000 [132].

TQ: Timer Questionnaire by Franzoi SL, 1989 [133].

SBA: Scale of Body Awareness by Hansell S, Sherman G, Mechanic D, 1991 [134].

QCC: Un Questionario di Consapevolezza Corporea (A Questionnaire on Body Awareness – Italian) by Lombardo C, San Martini P, Violani C, 1995 [136].

PBCS: Private Body Consciousness Sub-scale of the Body Consciousness Questionnaire by Miller LC, Murphy R, Buss H [135].

BPQ subscales: Body Perception Questionnaire (Awareness, Stress Response and Autonomic Nervous System Reactivity Subscales) by Porges SW, 1993 [139].

SBC: Scale of Body Connection by Price C, 2005 [74].

BVS: Body Vigilance Scale by Schmidt NB, Lerew DR, Trakowski JH, 1997 [141].

BAQ: Body Awareness Questionnaire by Shields S, Mallory M, Simon A, 1989 [142].

MHQ: Health Consciousness Subscale of The Multidimensional Health Questionnaire by Snell WE Jr, Johnson G, 1996 [143].

See Table 3 for criteria used to evaluate instruments in each category.

### Iterative Conceptual Review Process and Construct Refinement

The second aim of this study was to assess the understanding that underlies the constructs in the reviewed instruments independent from the original authors' statements. Four reviewers (JD, VG, WM, and CP) repeatedly reviewed the entire anonymized item pool extracted from all instruments and attempted to cluster and label the items according to domains and sub-domains. This conceptual review included all instruments included in the psychometric review as well as individual body awareness-related items from several instruments. Although excluded from the psychometric review for not providing subscales for body awareness and including only a few items regarding internal body sensations (Table 2), we decided to review the Kentucky Inventory of Mindfulness Skills (KIMS) [117], the Five-Facet Mindfulness Questionnaire (FFMQ) [125] and the Mindful Attention Awareness Scale (MAAS) [126] in the conceptual review step, as they have been widely used in the mindfulness literature, and aspects of mindfulness appear to constitute a key element of body awareness. Based on theoretical considerations, multi-disciplinary expert discussions parallel to formulating our operational definition (see above) and previous work by the authors we started with 7 domains: perceptivity of variations regarding pain, muscular tension, emotional/physiological state, and subtle body cues; emotional awareness/alexithymia; bias in sensation appraisal; mode of attention regarding labeling and immediate sensory experience; sensory versus affective discrimination; body-connection; and psychosomatic interconnectedness. The definition for these dimensions, their differentiation from and their relationships to each other were subjected to a continuous discussion among the reviewers leading to repeated reformulations. These multiple revisions of our initial model were followed by subsequent, renewed reviews of the entire item pool until an acceptable fit between items and model was reached. This iterative process continuously refined our operational definition into a conceptual model with explicit domains and sub-domains. Instead of presenting here our initial definitions and reformulations at every step of this process, we refer to the detailed, resulting dimensions and sub-domains with their definitions in the following section.

## Results

The systematic search yielded a total of 1,825 abstracts and identified 39 instruments related to body awareness. Twelve instruments satisfied our inclusion criteria for the psychometric review (Figure 1). Numerous instruments were exclusively developed to measure anxiety or to assess body image and self-objectification. 27 instruments were excluded (Table 1). The Body Awareness Rating Scale [127], [128] is an observer-based rating scale that judges the functionality of various visually observed movement patterns; it is exclusively used by specially trained physical therapists in Scandinavian countries, requires extensive training which, to our knowledge, is not available, translated or used outside these countries and had mixed results for inter-examiner reliability [129]–[131].

### Brief Description of the Twelve Measures Included in Psychometric Review

#### Body Intelligence Scale (BIS) [118]

Body intelligence was defined as “the awareness and use of bodily sensations to (a) support health and well-being, (b) supply information about environmental safety and comfort, and (c) enhance personal and spiritual development over a lifetime.” The scale development was described in detail, steps of which included the author's personal history, an immersion in ‘embodied writings’ in transpersonal psychology, the analysis of texts by body theorists and practitioners, and focus groups with body-centered psychotherapists, counselors and body practitioners. 200 items were field tested in healthy individuals and reduced to 31 according to exploratory factor loading on three subscales: energy body awareness (12): “awareness of energy inside and exterior to the physical body that signals safety and support, health and well-being;” comfort body awareness (10): “feelings of comfort with one's body and feeling of being ‘at home’ in the world;” and inner body awareness (9): “awareness of minor changes inside the body and the relationship of these felt changes to external circumstances.” The author provided data for internal consistency.

#### Body Responsiveness Questionnaire (BRQ) [23]

This 7-item instrument was designed to measure responsiveness to bodily sensations “broadening the construct of body awareness by emphasizing how bodily sensations are valued and treated and not just whether they are perceived.” The construct was based on the objectification theory of Fredrickson and Roberts (1997) and Yoga literature. An unpublished factor analysis in healthy women suggested two underlying factors: ‘Perceived disconnection between mental and physical processes’ and ‘trust in bodily sensations’. Yoga practitioners reported significantly greater body responsiveness compared to step aerobic students and a baseline comparison group. In cross-sectional data, the BRQ showed significant mediation between self-objectification and disordered eating attitudes. In an uncontrolled yoga trial, BRQ scores were positively associated with positive affect, satisfaction with life, and self-acceptance and negatively associated with negative affect. However, BRQ scores did not distinguish among women with different frequencies of yoga practice and were not sensitive to change with Yoga practice among experienced practitioners [23].

#### Body Awareness Measure (BAM) [132]

This 13-item instrument was designed to assess “psychotherapists' general awareness of their body in responses to the client and the client's material” as a way of managing counter-transference. Body awareness was defined as “pure sensory awareness which may or may not also have emotional or cognitive tones” and as “awareness of the body as experienced from within.” Scale development was explained by referring to limitations of prior instruments and by an exploration in a pilot sample of students. BAM was validated in a sample of 490 psychotherapy clinicians. Neither factor analysis nor sub-domains were reported. The author found no relation between therapists' BAM scores and measures of affect dysregulation or externalization of emotions and only small, non-significant inverse relations with measures of vicarious traumatization, possibly due to poor response rates. Therapists using somatic techniques scored significantly higher on BAM scores.

#### Timer Questionnaire (TQ) [133]

This 4-item instrument was developed to explore gender differences in body awareness and the influence of body esteem on body awareness. Body awareness was defined as “attention to the body” and “experience of the body during normal daily activity”. Distinguishing between attention to and concern for the body, the scale intends to measure the degree of awareness of the body, its perceived importance, the affect when “attending to important ‘body thoughts’”, and whether body awareness is specific (to certain body parts) or more global during a randomly sampled 10-minute recall. In male students scores were positively related to Body Esteem, whereas in females scores were positively related to beliefs about physical criteria in judging attractiveness. Reliability data were not available.

#### Scale of Body Awareness (SBA) [134]

This 4-item instrument was designed to assess the association between body awareness and medical care utilization among older adults. The authors viewed body awareness as a component of illness behavior rather than a body-mind process quality that can be independent of disease. Body awareness was defined as a “specific type of self-attention” or “introspectiveness” supposingly similar to the notion of private body consciousness developed by Miller.[135]. The instrument's items were meant to indicate the degree to which individuals think about the body and notice changes in the way the body feels and works. A one-dimensional factor structure was found in more than 1,000 older adults. Higher scores on the SBA showed a significant but minute association with the number of patient initiated clinic visits. Higher scores were associated with significant longitudinal decreases in self-assessed health; physical illness was cross-sectionally correlated with SBA scores but not with worsening scores over time.

#### Un Questionario di Consapevolezza Corporea (QCC) [136]

the authors defined body consciousness as interoception or “the capacity to supply discriminative responses in the presence of diverse visceral events” and as “sensori-motor fine tuning allowing a certain degree of control over these responses”. By expanding the scope of Miller's PBCS with including visceral perception, 30-items were created to evaluate “interoceptive ability” separate from (a) somatizisation and (b) hypochondriasis. Factor analysis in a student sample was incongruent with apriori subcales and resulted in a 3-factor model: ‘frequency of symptoms as a proxi for hypochondriasis’; ‘visceral perception’; and ‘general preoccupation with health’. Positive correlations between subscales and with Eysenck neurotizism scale were reported.

#### Private Body Consciousness Sub-Scale (PBCS) of the Body Consciousness Questionnaire (BCQ) [135]

This is one of the earliest, widely used and cited instrument attempting to measure body awareness. The authors extended a concept of public and private self-consciousness to awareness of the body in non-affective states. Starting with a set of items taken at face value, items dealing with pain or illness were omitted to avoid overlap with hypochondriasis, and items concerning strength, effectiveness, and grace of the body were added. Initial validation in student samples revealed three factors: ‘public body consciousness’, ‘private body consciousness’ and ‘body competence’. PBCS is the 5-item subscale for a “disposition to focus on internal body sensations”, “being aware of interoceptive feedback”, and being “sensitive to changes in bodily states”. The instrument has been used in a variety of patients i.e. with chronic pain [137]. PBCS scores do not correlate with social anxiety, hypochondriasis or emotionality. Scores were similar across different diagnostic groups and controls, supporting the construct as dispositional and not secondary i.e. to chronic pain. Validity and reliability were confirmed by multiple authors. Healthy young woman showed improved PBCS scores after a 7-week exercise program associated with improved fitness [138]. Higher scores were related to improved outcomes in hemodialysis patients [18].

#### ‘Awareness’, ‘Stress Response’ and ‘Autonomic Nervous System Reactivity’ subscales of the Body Perception Questionnaire (BPQ) [139]

This instrument was available online only. We were unable to obtain any supporting publication by the author regarding development or validation. However, Critchley et al. showed that scores on the awareness subscale of this instrument (45 items) as a “measure for self-rated bodily awareness” cross-sectionally correlated with gray matter volume in the right anterior insula of healthy subjects' cortices [32]. Also, BPQ scores could discriminate between groups of participants, who either used a desktop computer or an immersive version of the Cityscape Virtual Environment, the latter with head-mounted display and two 3D mice being “less perceptually aware of their bodies” [140].

#### Scale of Body Connection (SBC) [119]

This 20-item instrument was recently designed for use in body therapy and mind-body intervention research. A detailed description of scale development was provided including: initial item pool generation by the author based on clinical expertise and literature in body psychotherapy, body work therapy and allied fields; and testing for face and content validity by students and body work practitioners. The scale represents two independent dimensions confirmed by confirmatory factor analysis in a student sample: A) ‘body awareness’ is ”multi-faceted and involves sensory awareness, the ability to identify and experience inner sensations of the body (a tight muscle) and the overall emotional/physiological state of the body (relaxed, tense)”; B) ‘bodily dissociation’ is “characterized by avoidance of inner experience”. A study of women recovering from sexual abuse support the scale's reliability, validity and sensitivity to change in response to massage or body-oriented therapy [74].

#### Body Vigilance Scale (BVS) [141]

This 4-item inventory was “designed to assess attentional focus to internal bodily sensations” in the context of panic disorder referring to hypervigilant “conscious attention focused on internal bodily sensations and perturbations”, primarily to “interoceptive threat cues”. Three items assess the “degree of attentional focus, perceived sensitivity to changes in bodily sensations and the average amount of time spent attending to bodily sensations”. A fourth question assesses attention ratings to 15 sensations that summarize the DSM-IV physical symptoms for panic attacks. Field testing was done with students, community samples, and panic disorder and phobia patients. Factor analyses revealed a one-dimensional factor structure for a “stable disposition that may, for some individuals, act as a risk factor for the development of anxiety pathology vis-a-vis increased perception of bodily sensations”. BVS scores were associated with a history of panic and with anxiety sensitivity, anxiety and depression symptoms but not with trait anxiety (STAI). Score changes after cognitive-behavioral therapy were associated with reduced anxiety.

#### Body Awareness Questionnaire (BAQ) [142]

This is a self-report scale for “measuring beliefs about one's sensitivity to normal, non-emotive body processes” widely used in a variety of settings. Referring to the limitations of previous instruments that only measure “physical symptoms characteristic of illness or other somatic complaint” or “sensitivity to emotion related bodily responses,” scale development began with generating a 52-item item pool meant to represent the “the domain of reported awareness of normal bodily processes not typically associated with emotion or with somatic complaint.” After testing for face validity and administration to several student samples, an 18-item version was developed. Factor analysis revealed four sub-domains not scored separately: ‘Note response or changes in body process’, ‘predict body reactions’, ‘sleep-wake cycle’ and ‘prediction of the onset of illness’. Multiple studies by various authors strongly support reliability, convergent and discriminant validity. Sensitivity to change was not assessed.

#### Health Consciousness (HC) subscale of The Multidimensional Health Questionnaire (MHQ) [143]

The 5-item health consciousness subscale is one of 20 subscales in a questionnaire developed to assess psychological correlates of health behavior and refers to “an awareness of one's health as a measure of the tendency to think about and reflect about one's health. People who endorse these items are those who think about the status of their physical health, and who in general are reflective about the nature of health and wellness of their body.” The authors state that the basis for this instrument is 1) previous research indicating that “individual psychological dispositions clearly play an important role in mediating men's and women's health behaviors,” and 2) a previously developed measure “Health Orientation Scale” with a HC component. HC was used in students and elderly Italians. A principal component analysis HC loaded on a factor labeled ‘Health Management Factor’. Preliminary reliability or validity data were provided.

### Summary of Results of Psychometric Evaluation

Table 4 provides detailed information about the psychometric properties of each instrument. In summary, few of the instruments have strong psychometric properties for rigorous research or clinical applications. Only two instruments have been used more than a few times. Regarding their dimensions, the BAQ [142] is one-dimensional and explicitly excludes attention to pain sensations and emotions. Similarly, the PBCS [135] with its 5 items assesses primarily a single dimension.

Two instruments have been applied to both research and clinical use. Two instruments (BAQ, PBCS) fulfilled a high standard for reliability and four (BAQ, BVS, PBCS, SBC) for validity (characterized with three or more “*”s in Table 4). Five instruments were administered in patients but only one in patients with pain (PBCS). With three instruments (BAQ, BIS, SBC) we found evidence of systematic construct development. If we add up the psychometric “stars” each instrument qualified for (including one for more than a few studies it was used in and one for clinical use, thus allowing for a maximum of 23 “stars”) we find the BAQ (18) as the strongest instrument, followed by PBCS (15), BVS (15), and SBC (13). Yet two of these instruments have only 4 or 5 items, which brings up the question of which dimensions of the body awareness construct the reviewed instruments assess.

### Emerging Key Dimensions of the Body Awareness Construct

This question is addressed with a conceptual review of the questionnaire items to determine which domains of body awareness were queried. As described above, a theoretical model for the body awareness construct was developed in a parallel iterative process from a preliminary operational definition to a more refined model. The following four inter-related dimensions emerged (Table 5):

**Table 5:** Dimensions of Body Awareness (further details: see in ‘Results’).

Dimension	Sub-Domain	Explanation
1) Perceived Body Sensations	A) Sensations of distress, worry, pain and tension	Ability to note changes in body processes, to identify inner sensations (e.g. a tight muscle, fatigue, warmth, pain) and discern subtle bodily cues indicating varying functional states of the body or its organs and the emotional/physiological state of the body (relaxed – tense).
	B) Sensations of wellbeing	Sensory and affective aspect of sensations.
	C) Neutral/ambiguous sensations	
	D) Affective aspect of sensation: Bothersomeness i.e. of pain	
2) Attention Quality	A) Intensity: Actively paying attention (incl. exaggerated focus) vs. ignoring and suppressing perceptions.	A) Bi-polar continuum from paying attention towards sensations (understood as *active* response to the perception of sensations) to distracting avoidance, ignoring and suppression of perceptions. Active focus can be involuntarily reactive as well as intentional (“mindful”). Intensity also reflects the importance of one's body sensations to the individual.
	B) Self-efficacy in attention control	B) Confidence in the ability how well one can focus on a sensation, sustain focus and control the mode of attention.
	C) Mode: thinking/labeling vs. experiencing the present-moment immediacy of sensations	C) Bi-polar continuum from reflective, mental, analytical, thinking, labeling, ruminating mode to non-judgmental, immediate, felt sensory awareness, mindful presence (includes kinesthetic sense).
3) Attitude	A) Trusting	General (trait) bias in appraisal/interpretation of sensations: Variance in how we relate to bodily cues: (A) trust and viewing sensations as helpful for decision-making and sense of self;
	(B) Catastrophizing	(B) catastrophizing and worry.
4) Mind-Body Integration	A) Emotional awareness	A) Awareness of physical sensations in emotions as their sensory aspect (“somatic markers”).
	B) Overall felt sense of embodied self vs. feeling disconnected.	B) Bi-polar continuum from feeling embodied (with awareness of interconnectedness of mental, emotional, and physical pro-cesses) to a sense of alienation from one's body.

1. Perceived body sensations or the ability to note changes in body processes, to identify inner sensations (e.g. a tight muscle, fatigue, warmth, pain) and to discern subtle bodily cues indicating varying functional states of the body or its organs and the emotional/physiological state. This dimension is the primary sensory, physiological aspect of body awareness with its early, mostly pre-conscious appraisal or affective “coloring” of that sensation. It is subdivided into four sub-domains: A) sensations of distress, worry, pain and tension (e.g. “I am aware of tension in my muscles”); B) sensations of wellbeing (e.g. “I feel my feet warming up when I relax”); C) neutral or ambiguous sensations (e.g. “I notice changes in how my body feels”), and D) the affect aspect of sensation or bothersomeness i.e. of pain (e.g. “How much does your back pain bother you?”). The affect component of a body sensation is here understood as determined by the early preconscious (i.e. with acute pain) or the secondary, evaluative appraisal.
2. Quality of attention with 3 sub-domains: A) The intensity of attention along a bi-polar continuum from paying attention to sensations (seen as an active response to the perception of sensations and including exaggerated attention) on one end to distracted avoidance, ignoring and suppression of perceptions on the other end (e.g. “I distract myself from uncomfortable body sensations.”). This reflects the importance of body sensations to the individual and does not reflect whether this active focus is involuntarily reactive or intentional (“mindful”). B) The self-efficacy of attentional control or the individual's confidence in the ability to focus on a sensation and sustain or control the mode of attention (e.g. “I can move my attention to different parts of my body.”). C) The mode of attention or how an individual pays attention to a sensation, whether her attention is more in a mode of (a) either thinking about, reflecting on, judging, analyzing one's sensation with the extreme of ruminating (e.g. “How much do you think about how your body feels?”) or (b) non-judgmental, immediate experience and sensory awareness of that sensation (e.g. “When I am walking, I deliberately notice the sensations of my body moving.”), with mindful presence as the polar opposite to rumination. This dimension reflects a process component of body awareness, the active act of paying attention, which modifies, filters, or augments the sensory input from the body. Self-efficacy can be seen as a relatively stable trait but one that can potentially be modified by learning how to control the intensity and quality of one's attention.
3. Attitude of body awareness refers to two domains describing how individuals relate to bodily cues: (A) trusting or viewing bodily sensations as helpful for decision-making and sense of self (e.g. “It helps to listen to my body”) and (B) worry and catastrophizing (e.g. “Feelings from inside my body make me worried about diseases.”). This dimension is understood as a general trait-like bias towards appraisal of the perceived sensation and is a further modifier of the perceived sensations, a second key trait, relatively stable but potentially modifiable by targeted, therapeutic interventions. The effect of this trait on perceived sensations is thought to be mediated by the mode of attention (2C).
4. Awareness of mind-body integration can be experienced as subjective evidence in two sub-domains: A) as emotional awareness, the awareness that certain physical sensations are the sensory aspect of emotions (as in the theory of “somatic markers” [39], [63])(e.g. “I notice that my breathing becomes shallow when I get nervous”) or B) as an overall felt sense of an ‘embodied self’, representing a second-order perception of sensations that contains within it a felt sense of the interconnectedness of mental, emotional, and physical processes as opposed to a disembodied sense of alienation and of being disconnected from one's body [38], [56], [144] (e.g. “I feel at home in my body.”).

### Summary of Conceptual Review

Table 6 presents the dimensions and sub-domains, which we determined as underlying the items in the reviewed instruments. The BAQ stands out as the only instrument of which the entire item pool (18 items) relates to only one dimension, that of subtle body cues and of perceived body sensations. Similarly, the much shorter 5-item PBCS mostly measures one single sub-domain, namely sensations of distress, worry and tension. All other instruments measure multiple dimensions of the body awareness construct.

**Table 6:** Number of items that fall into predefined dimensions and sub-domains for each questionnaire.

Domains	Sub-Domains	BIS	BRQ	BAM	TQ	SBA	QCC	PBCS	BPQ	SBC	BVS	BAQ	MHQ
1 Perceived Body Sensations	1A Sensations of distress and worry, pain and (muscle) tension	2 +2A: 1	0	+1B: 1 +2A: 1	0	0	17 +2A: 4	3 +1C: 1	69 +1C: 5 +2B: 2	1 +1C: 1 +4A: 3	0	3	0
	1B Sensations of wellbeing	1 +1D: 1 +3: 1	0	+1B: 1	0	0	0	0	0	1 +4A: 1	0	0	0
	1C Neutral/ambiguous sensations	6 +3: 1 +4B: 1	0	1 +2A: 8	1	1	4	+2A: 1	+1A: 5	+1A: 1	0	15	+2A: 5
	1D Bothersomeness of symptoms, i.e. pain	+1B: 1 +3: 1	0	0	1	0	0	0	0	0	0	0	0
2 Attention Quality	2A Intensity; actively paying attention versus ignoring and suppressing perceptions	3 +1A: 1 +3: 1	1 +3: 1	+1C: 8 +1A: 1	1 +3: 1	+2C: 1	+1A: 4	+1C: 1	0	2	4	0	+1C: 5
	2B Self-efficacy	1	0	0	0	0	0	0	+1A: 2	0	0	0	0
	2C Mode: thinking/labeling versus experiencing immediacy of sensations	0	0	0	0	1 +2A: 1	0	0	0	0	0	0	0
3 Attitude to Body	3A Trusting	2 +1B: 1 +1C: 1 +1D: 1 +4B: 1	3 +2A: 1 +4B: 1	0	+2A: 1	0	0	0	0	2 +4A: 1	0	0	0
	3B Catastrophizing:	2 +2A: 1	1										
4 Mind-Body Integration	4A Emotional awareness	1	0	2	0	0	0	0	0	2 +1A: 3 +1B: 1 +3: 1	0	0	0
	4B Felt sense of an embodied self vs. feeling disconnected	1 +1C: 1 +3: 1	+3: 1	0	0	0	0	0	0	3	0	0	0
	Conceptual mismatch (items not about body awareness)	4	0	0	0	0	5	0	6	3	0	0	0
	Sub domains covered (out of 11)	10	4	5	4	3	3	3	3	7	1	2	2

Items fitting in 2 domains are included as “+” with the respective second domain. Acronyms are explained in Table 4.

However, their items frequently are unable to discriminate between distinct dimensions and, therefore, do not fall into single domains. This lack of discrimination reveals a limitation of the instruments. Multiple reformulations of the dimensions and sub-domains aided in simplifying the ordering of items but could not overcome this limitation.

Intriguingly, only one instrument dedicated to body awareness (SBA) included a single item to measure the quality or mode of attention, mindfulness versus labeling thought (2C), that seems to make all the difference between body awareness being adaptive or maladaptive. If the quality of attention was addressed at all, then the instruments ask only about the thinking or labeling end of the spectrum and not about the other end characterized by mindfulness or immediate present-moment awareness. Instruments specifically designed to measure mindfulness, such as the uni-dimensional Mindful Attention Awareness Scale (MAAS) [126], the Kentucky Inventory of Mindfulness Skills (KIMS) [117], and its successor the Five-Facet Mindfulness Scale (FFMS) [70], include items regarding awareness specifically of internal body sensations but do not provide subscales for body awareness (Table 7). The MAAS includes a single item (item 5), which is missing from the factor analysis by Baer [70] without explanation, might load on the ‘observing’ facet of mindfulness and was included in the conceptual review. KIMS and FFMQ contain subscales labeled “Observing”; however, these subscales include items for the awareness of thoughts and external stimuli (sights, sounds, smells) that disqualified them from our psychometric review. Half of the 12 ‘observe’ items in the KIMS address body awareness (items 1, 5, 9, 13, 17, 21). The other half refer to observations of thoughts, external stimuli or cognition regarding emotions. Also, item 22 in the ‘describe’ facet addresses body sensations. Therefore, 7 items qualified for our conceptual review. With the exception of items 1 and 5, four of the ‘observe’ items are included in the FFMQ (four of eight, again half of the ‘observe’ items) with the addition of item 22. The KIMS is the only instrument that includes four items (three of which are included in the FFMQ: items 1, 6, 15) querying the immediate present moment awareness of bodily sensations.

**Table 7:** Instruments with individual body awareness-related items (without subscale).

Domains	Sub-Domains	KIMS	FFMQ	SSAS	SIPOAS	MAAS	BSIQ	EDI-2/3	SAQ	APQ	PBSQ
	Number of body awareness-related items	7	5	6	10	1	8	4	5	10	5
1 Perceived Body Sensations	1A Sensations of distress and worry, pain and (muscle) tension	0	0	1	0	0	0	1	1	4	2
	1B Sensations of wellbeing	0	0	0	0	0	0	0	0	1	0
	1C Neutral/ambiguous sensations	1 +2A: 4 +4A: 1	+2A: 3 +4A: 1	2	0	0	0	0	+4A: 1	4	1
	1D Bothersomeness of symptoms, i.e. pain	0	0	3	0	0	0	0	0	1	0
2 Attention Quality	2A Intensity; actively paying attention versus ignoring and suppressing perceptions	+1A: 4	+1A: 3	0	0	1	0	0	2	0	0
	2B Self-efficacy	0	0	0	0	0	0	0	0	0	0
	2C Mode: thinking/labeling versus experiencing immediacy of sensations	0	0	0	0	0	0	0	0	0	1 +3A: 1
3 Attitude to Body	3A Trusting	0	0	0	1 +1A,4A: 1 +4A: 1	0	+3B: 8	2	0	0	+2C: 1
	3B Catastrophizing	0	0	0	0	0	+3A: 8	0	0	0	0
4 Mind-Body Integration	4A Emotional awareness	+1C: 1	+1C: 1	0	4 +1A: 1 +1B: 1 +1A,3A: 1 +3A: 1	0	0	0	+1C: 1	0	0
	4B Felt sense of an embodied self vs. feeling disconnected	0	0	0	0	0	0	1	0	0	0
	Conceptual mismatch (item about ability to find words only)	1	1	0	0	0	0	0	0	0	0
	Sub domains covered (out of 10)	3	3	3	2	1	2	3	4	4	4

Items fitting in 2 domains are included as “+” with the respective second domain. Acronyms are explained in Table 2.

All body awareness-related items of the BDI and HAM-A fit into sub-domains of one dimension: ‘sensations of distress and worry; (1A in table 5); the SSAS includes two items for ‘neutral sensations’ (1C), and three for ‘bothersomeness of symptoms’ (1D).

BIS, BAM and SBC assess emotional awareness (4A), SBC, BIS and BRQ assess the felt sense of embodiment, one single item of the BIS assesses self-efficacy in attention control (2B), and two instruments assess sensations of wellbeing (2B). The broadest coverage of our dimensions and the total of eleven sub-domains are provided by BIS with 10, SBC with 7, and BAM with 5 sub-domains, respectively.

## Discussion

We reviewed existing self-report instruments for the measure of body awareness and assessed their psychometric properties. Although this review found that the concept of body awareness is widely used, it also revealed important limitations to the current approaches in understanding the meaning of measures of body awareness:

First, there is no widely accepted unifying measurement definition. Numerous instruments were exclusively developed to measure anxiety or assess body image. Body awareness is considered by most authors to be a single, mostly undifferentiated construct. However, among uni-dimensional definitions of body awareness, the dimensions queried by the items vary considerably across instruments and represent different aspects of the construct.

Second, according to the dimensions and sub-domains emerging from our qualitative review, in most instruments, item wording does not clearly discriminate between dimensions reflecting a lack of systematic measurement development.

Third, most current definitions of body awareness appear to be dominated by the concern that heightened body awareness necessarily leads to somatosensory amplification, worsens symptoms of anxiety and hypochondriasis, and is maladaptive for clinical outcomes, such as pain. Accordingly, most measures list symptoms that are expected to be appraised by patients as uncomfortable or threatening. Currently, validated measures for body awareness are not able to discern between (a) anxiety-related hypervigilance toward pain and other physical sensations with catastrophizing interpretation bias and (b) a non-judgmental, meditative, ‘mindful’ awareness of these sensations. Thus, the existing instruments perpetuates the persisting confusion about the benefits of focused attention either away (distraction) or toward internal physical sensations. For the investigation of any therapeutic approaches to chronic pain and other conditions that claim to enhance body awareness, such measures cannot suffice.

Fourth, key dimensions are missing in most of the reviewed instruments. Only two instruments have strong psychometric properties *and* were used in more than a few studies: BAQ and PBCS. Regarding the dimensions emerging from this review, the BAQ [142] is uni-dimensional and explicitly excludes attention to pain sensations and emotions. As we are interested in a ‘mindful’ focus of body awareness on i.e. uncomfortable physical sensations, pain in particular, or on the sensory aspects of emotions, most key dimensions according to our understanding of body awareness are not covered by the BAQ. The PBCS [135] with its 5 items assesses primarily a single dimension. It has been suggested that benefits from mind-body interventions may be derived from an uncoupling of a ‘narrative self-focus’ from an ‘experiential awareness focus’ [109]; non of the reviewed questionnaires is able to discern these qualities of body awareness. Newer and therefore less frequently used or validated measures (BIS [118], BRQ [23], SBC [145], and Sherman (personal communication)) were developed and/or validated for specific patient groups. These instruments are missing several important dimensions of the body awareness construct without which it remains unclear how the concept of body awareness may aid in the patient evaluation and treatment of major medical illnesses. Thus these scales are limited by their focus on patients with specific diagnoses or lack a more systematic and comprehensive, multidimensional construct development.

In summary, we provided a comprehensive review of existing instruments with their psychometric properties for the measure of body awareness. In parallel, we assessed the theoretical constructs underlying existing measures of body awareness by using an iterative process to synthesize the literature and the current understanding of body awareness into a proposal for a multi-dimensional construct. Several instruments demonstrate strong psychometric properties (BAQ, PBCS). For lack of a better instrument available today, the conceptual review in conjunction with the psychometric review can aid researchers in selecting currently available measures for particular dimensions of body awareness. However, not one single reviewed instrument nor a compilation of several of these are able to satisfactorily cover all the dimensions of our construct. There is a great need to develop an instrument that overcomes these limitations. We provide a detailed description of the construct's dimensions and sub-domains. This can be used as the theoretical framework from which the systematic development of a multi-dimensional body awareness measure can begin.

None of these self-report measures has been validated against an objective measure. As self-report awareness measures remain necessarily limited, options for new performance and experimental measures need to be explored in future research. Presently, the most-commonly applied objective measure for the construct is a performance measure used in interoception research to assess heart rate detection accuracy [31], [33], [146], [147]. However, it measures a single element of interoception focusing on the heart. Although heart beat has the advantage of being less susceptible to voluntary manipulations when compared i.e. with respiration, none of the body awareness training approaches we reviewed focus on heart rate perceptions. Several of these techniques, i.e. meditation, focus and train awareness of breathing rather than heart beat. Although various heart rate detection test designs (including the systemic application of isoproterenol) appear rather convincing in the context of anxiety and emotional arousal, these tests are unable to distinguish between groups of experienced meditators and matched controls [148], [149], [150] or to assess more subtle changes in relaxed subjects [151]. To this date, it remains unclear whether any mind-body approach, meditation or others enhance forms of body awareness other than heartbeat detection, or whether increases in body awareness translate to all of its aspects, or whether these approaches enhance body awareness at all. Respiratory interoception has been assessed by measuring respiratory resistive load detection primarily in healthy subjects [152] or patients with asthma [153] but not in meditators. An interoceptive respiratory load biofeedback training did not improve asthma symptoms [153], however a study using yoga respiration training in healthy subjects showed improved respiratory interoception [108]. Gastrointestinal (GI) distension has been used to study gastrointestinal interoceptive sensitivity [154], [155]. Sensitivity is increased in patients with irritable bowel syndrome in conjunction with hypervigilance, and a systematic desensitization was associated with improved symptoms [156], [157]. In addition to further studies of single-modality of interoception, objective measures of trans-modal interoception might be developed in the near future, for which currently no gold standard is available. Similar to the research of pain, in the immediate future broader clinical research in the field of body awareness will also have to rely on self report. Once self-report measures can be validated against objective measures, they will remain important tools for the clinical setting outside of an experiment laboratory.

The immediate next step would be to further define the construct and its dimensions and sub-domains through qualitative research by conducting focus groups with both therapists of approaches addressing body awareness and patients undergoing such therapies. The same focus groups can review a pool of items collected from prior instruments and aid in developing items that clearly distinguish between dimensions. Subsequently, this item pool needs to be evaluated by appropriate psychometric tests in patient samples to be reduced to a questionnaire that allows us to make progress in this important field of psychosomatic research.
